# Quality Characteristics, Nutraceutical Profile, and Storage Stability of Aloe Gel-Papaya Functional Beverage Blend

**DOI:** 10.1155/2014/847013

**Published:** 2014-08-18

**Authors:** Pushkala Ramachandran, Srividya Nagarajan

**Affiliations:** Food Science, Technology and Nutrition Division, Department of Home Science, Sri Sathya Sai Institute of Higher Learning, Anantapur, Andhra Pradesh 515001, India

## Abstract

*Aloe vera* gel, well known for its nutraceutical potential, is being explored as a functional ingredient in a wide array of health foods and drinks. Processing of exotic fruits and herbal botanicals into functional beverage is an emerging sector in food industry. The present study was undertaken to develop a spiced functional RTS beverage blend using Aloe gel (AG) and papaya. Aloe gel (30%), papaya pulp (15%), spice extract (5%), and citric acid (0.1%) were mixed in given proportion to prepare the blend with TSS of 15 °Brix. The product was bottled, pasteurized, and stored at room temperature. The quality characteristics and storage stability of the spiced beverage blend (SAGPB) were compared with spiced papaya RTS beverage (SPB). Periodic analysis was carried out up to five months for various physicochemical parameters, sugar profile, bioactive compounds, microbial quality, instrumental color, and sensory acceptability. The SAGPB exhibited superior quality characteristics compared to SPB both in fresh and in stored samples. The SPB was acceptable up to four months and SAGPB for five months. The results indicate that nutraceutical rich AG could be successfully utilized to develop functional fruit beverages with improved quality and shelf life.

## 1. Introduction

Fruits and vegetables have always had an elite status among the health foods. However, due to their short shelf life, processing into preserved products becomes essential. Fruit juices and beverages are one such processed product which is convenient to use and also help meet the daily requirement of fruits and vegetables in the diet. Healthy beverages, particularly those that offer functional ingredients such as botanicals, minerals, and antioxidants, are increasing in demand. New product introductions in the health drink and fruit juice categories were found to reach over 700 new offerings in year 2003, up 40% over 2002 [1]. Functional beverage sector has been reported to be the fastest growing segment [2]. Fruit based functional beverages with refreshing flavors and tastes are being preferred over aerated drinks by the health-conscious consumers, in particular. Further value enhancement is feasible with addition of nutraceuticals. Nutraceuticals from botanical origin have a great scope to offer varied health benefits such as improved antioxidant profile and protection against various chronic metabolic diseases.

Papaya is one of the main tropical fruits produced in India. India was reported to be the largest papaya producer (2.7 MT) in the world in 2008 [3]. Apart from its luscious taste and attractive color, papaya is a rich source of antioxidant nutrients such as carotenoids, vitamins C and E, and flavonoids; the B vitamins, folate, and pantothenic acid; and the minerals, potassium, magnesium; and fiber. Together, these nutrients are known to promote the health of the cardiovascular system and protect against colon cancer [4]. Hence, for the present study, papaya was selected for preparation of functional fruit beverage.

As the nutraceutical ingredient,* Aloe vera* (*Aloe barbadensis* Miller) gel was selected. This herbal botanical is well known for its numerous therapeutic effects. It is also being studied widely in recent years as a valuable ingredient in foods [5]. The Aloe gel is transparent slippery mucilage containing bioactive polysaccharides, mainly partially acetylated glucomannans. It is also a good source of vital nutrients [6].

New products combining the unpalatable Aloe gel juice with other health foods particularly antioxidant rich fruits such as berries, peaches, and their extracts has been produced. Its blend with tropical fruits such as papaya, however, remains to be explored. Spices are another group of botanicals that are being increasingly used for preparing appetizer drinks and also as an aid to mask undesirable flavor in beverages. Spices are also well known for their antimicrobial and antioxidant activities [7, 8] as well as for their therapeutic value [9, 10]. A combination of above two botanicals with papaya is proposed to result in a functional beverage with enhanced nutritional and nutraceutical value.

With the above facts in view, in the present study, a spiced Aloe gel-papaya blend functional beverage was developed and its physicochemical characteristics, antioxidant potential, microbial quality, and sensory acceptability were evaluated. The shelf life quality on storage for five months was also studied.

## 2. Materials and Methods

### 2.1. Experimental Materials

Uniformly ripened papaya of uniform size and shape was purchased from the local market of Anantapur. The* Aloe vera* leaves were obtained from the University garden. Food grade citric acid, sugar, salt, and other spices were procured in one lot from the local market.

### 2.2. Preparation of Spiced Papaya Beverage (SPB)

Papaya fruits were thoroughly washed under running tap water, peeled, and cut into small pieces. The pieces were finely blended to obtain papaya pulp, which was filtered through a muslin cloth. Sugar syrup (20%) was prepared and blended with 15% of the obtained papaya pulp. Spice extract was prepared by boiling aniseed, ginger, and pepper in water for few minutes. This was added to the papaya pulp-sugar syrup blend at 5% level. Citric acid was also added at 0.1% level to achieve the desired acidity level recommended for beverages. The resultant spiced papaya RTS beverage (SPB) was again blended and heated for a few minutes in low flame. The SPB was then hot-filled into presterilized glass bottles, screw capped, and pasteurized for about 15 minutes at 80–90°C followed by immediate cooling.

### 2.3. Development of Spiced Aloe Gel-Papaya Beverage Blend (SAGPB)

The fresh green* Aloe vera* leaf was taken for the extraction of Aloe gel juice. The Aloe gel was removed carefully by the hand filleting method from the leaf. The gel was washed 3-4 times in distilled water, hand crushed, blended, and filtered through muslin cloth. Similar procedure was followed for the preparation of spiced Aloe gel-papaya blend beverage (SAGPB) as SPB, except that in SAGPB, Aloe gel juice obtained was added at different levels of 10%, 20%, and 30% to prepare 10 SAGPB, 20 SAGPB, and 30 SAGPB, respectively. This was followed by addition of other ingredients, hot filling, pasteurization, cooling, and storage.

The developed beverages were initially evaluated for sensory acceptability in terms of color, flavor, taste, consistency, and overall acceptability. The Aloe gel enriched beverage found best in terms of the above mentioned parameters was selected for further storage analysis, along with the control beverage SPB. The selected beverages were stored at room temperature (28 ± 2°C) in dark up to five months. Analysis for various parameters was carried out on 0 d and after 45 d, 90 d, 120 d, and 150 d of storage. The beverage was evaluated for physicochemical characteristics, phytochemicals, and microbial and sensory quality.

### 2.4. Analysis of Physicochemical Characteristics

The samples were analyzed for various physicochemical parameters. The pH of the samples was read with a calibrated pH meter (Elico India L1-120 model). Titratable acidity was measured using 0.1 N NaOH and expressed as percentage citric acid; total soluble solids (in °Brix) determined using digital hand refractometer (Atago, Japan) and nonenzymatic browning (NEB) estimated by measuring optical density (OD) at 440 nm. Total and reducing sugars were determined by the Lane and Eynon method using Fehling's reagent. All these procedures were carried out as described by Ranganna [11].

### 2.5. Instrumental Color

For the estimation of color, test sample was filled in a 3 inch diameter petri dish. The color was measured using a color reader (Konica MINOLTA CR-10) and expressed as Hunter* L* ^∗^ *a* ^∗^ *b* ^∗^ units.* L* ^∗^ indicates luminosity or brightness,* a* ^∗^ corresponds to greenness (−)/redness (+), and* b* ^∗^ corresponds to blueness (−)/yellowness (+).

### 2.6. Phytochemical Analysis

A direct colorimetric method as given by Ranganna [11] was used for the estimation of vitamin C content. Methanol extractions of fresh samples were carried out using a modified method of Banerjee et al. [12]. These extracts were used for the estimation of total flavonoids and total polyphenols. Aluminium chloride method [13] was used for determination of flavonoids and the content expressed as mg quercetin equivalents (QE) in 100 mL of beverage. Total polyphenols were determined using Folin-Ciocalteu procedure [14] and the total phenolic content was expressed as gallic acid equivalents in milligrams per 100 mL of beverage.

### 2.7. Microbial Analysis and Sensory Acceptability

Total bacterial count (TBC) and yeast and mold counts (YMC) of the samples were determined by standard plate count method and expressed as log CFU mL^−1^.

The sensory acceptability of the samples was evaluated by a group of 20 untrained female panel members between 18 and 40 years of age, selected from the authors' university. The samples were randomly coded and served at 15°C in 50 mL aliquots to the panelists, along with plain water. The panelists judged the samples separately for unbiased evaluation of the sensory attributes. They were asked to rate the samples for various sensory attributes, namely, color, flavor, taste, consistency, and overall acceptability, on a 5-point hedonic rating scale with 5 and 1 indicating the highest and lowest scores, respectively. The samples were evaluated at the end of each storage interval and the mean scores were reported.

### 2.8. Statistical Analysis

Data obtained for the various parameters were expressed as mean values ± standard deviations of at least three replications. Statistical analyses were performed using SPSS (Statistical software Student Version 16.0, Chicago, IL, USA). Data was analyzed using one way ANOVA. Statistical difference between the means was determined using Duncan's multiple range test (DMRT) with the confidence limits set at *P* < 0.05 (95%).

## 3. Results and Discussion

### 3.1. Raw Material Characterization

Papaya fruit and fresh Aloe gel juice used in the present study were first characterized for few important parameters. Papaya pulp was found to have moisture content of 86.5% and TSS of 15 °Brix. Aloe gel recorded a higher moisture content of 98.9% and low TSS of 0.08 °Brix. Acidity of papaya pulp was also higher (0.33%) compared to Aloe gel (0.05%). A higher value of nonenzymatic browning was recorded in Aloe gel (0.09), whereas, that of papaya pulp was found to be 0.04. Papaya pulp was found to contain a good amount of vitamin C (46 mg/100 g). For Aloe gel, the vitamin C content was found to be 1.56%. Papaya pulp recorded 24.7 mg/100 g, 9.9 mg/100 g, and 14.8 mg/100 g of total sugars, reducing sugars, and nonreducing sugars, respectively, and the corresponding values for Aloe gel were 1.85, 0.03, and 1.82, respectively.

The various beverage blends of Aloe gel and papaya formulated were initially evaluated for sensory acceptability (Table 1). With regard to color, 30 SAGPB recorded significantly higher score of 4.78 compared to control (4.57). This could be attributed to the appealing brighter color of 30 SAGPB. Similarly, in terms of taste and flavor, 30 SAGPB recorded significantly higher scores compared to control. The lower score for control sample could be due to its predominantly sweet taste which was not well appreciated by the panelists when given in the generally consumed portion size (150 mL). A more balanced taste was witnessed in the AG enriched samples, which was found to increase with increase in percentage of AG. Slightly lower scores obtained for AG enriched samples for consistency could be due to its comparatively thinner consistency compared to control. The overall acceptability scores indicated 20 SAGPB and 30 SAGPB to be highly acceptable with scores of 4.64 and 4.71, respectively, significantly higher than 10 SAGPB and control. A major problem with commercial Aloe gel juice and beverages is adverse/bitter taste, which could be successfully avoided in the present study with suitable processing operation, with addition of spices and blending with papaya. Literature studies on Aloe gel administration to human subjects recommend about 30 mL consumption per day. Since the average consumption of a beverage for an individual could be about 100 mL and also because addition of AG at higher levels was more acceptable, 30 SAGPB was chosen for further storage studies.

### 3.2. Storage Stability Studies

The control spiced papaya beverage (SPB) and 30% Aloe gel-papaya spiced beverage blend (SAGPB) were periodically analyzed for various parameters on storage and results are discussed in the following section.

The physicochemical characteristics of the stored samples are given in Table 2. The initial titratable acidity of the samples was found to be 0.27%. With an increase in storage time, the acidity was found to increase. This could be attributed to the decomposition of fermentable substrate, especially, carbohydrates in the fruits and added sugar, thereby increasing the acidity [15]. Significantly (*P* < 0.05) lower acidity was recorded in SAGPB throughout the storage period. SPB was found to exhibit a higher increase in acidity to 0.62%, compared to the acidity value of 0.54% in SAGPB, after 150 d of storage. Similar increase in acidity on storage has also been reported in other beverages such as jamun [16] and other fruit flavored drinks [15].

Total soluble solids on the initial day were similar in SPB (15.6 °Brix) and SAGPB (15.7 °Brix), respectively (Table 2). An increase in TSS was observed on storage. Similar to titratable acidity, SPB recorded a higher TSS throughout storage compared to SAGPB. A significantly (*P* < 0.05) lower TSS of 18.7 °Brix was recorded in the SAGPB compared to SPB (25.2 °Brix). The increase in TSS observed on storage could be due to the conversion of polysaccharides to sugars. This could have also led to an increase in total and reducing sugar content on storage. The SPB beverage recorded an increase in total sugar content from 16.7% on the initial day to 29% at the end of storage period. This increase was lesser in case of SAGPB from 17% to 24.5% from 0 d to 150 d of storage. Similarly the initial reducing sugars content of the beverages was found to range from 6.7% to 6.9% which increased to 11% and 9.5% for SPB and SAGPB, respectively. An increase in reducing sugars has also been reported in case of whey-based banana herbal beverage during storage. This is attributed to the conversion of nonreducing sugars to reducing sugars [17].

The instrumental color of the samples was determined in terms of the* L* ^∗^,* a* ^∗^, and* b* ^∗^ values. In the papaya beverage, the predominant color is orange-red and, hence, +*a* ^∗^ value which denotes redness is the most important parameter, followed by +*b* ^∗^ which denotes yellowness. A higher* a* ^∗^ value of 2.36 was recorded in the SAGPB compared to SPB (1.63) on the initial day (Table 2). A degradation of red color was witnessed on storage for both the samples. The SAGPB samples could maintain higher* a* ^∗^ scores of 1.3, significantly higher than SPB (0.61) by the end of storage period. A decreasing trend was also witnessed in* b* ^∗^ values from 9.23 to 7.83 in SPB and from 11.9 to 9.3 SAGPB, from the initial to final day of storage, respectively. The SAGPB samples successfully maintained significantly higher* b* ^∗^ value during storage, thereby resulting in a brighter and more acceptable product.

Another important parameter critical during storage is nonenzymatic browning (NEB) which has been reported to be caused due to Maillard reaction and degradation of pigments. An increase in NEB on storage was seen in both the samples, though the extent of browning was significantly higher in the SPB samples compared to SAGPB (Table 2). The Aloe gel enriched beverage (SAGPB) recorded 35% lower NEB which could be attributed to the beneficial effect of Aloe gel addition. Aloe gel has been reported to have an antibrowning functionality when applied as a coating on whole [18] and minimally processed fruits [19].

The phytochemicals evaluated in the beverages on storage include vitamin C, total polyphenols, and total flavonoids. The results of their changes on storage are presented in Table 3. The initial content of vitamin C recorded in the samples on the initial day was 5.22 mg/100 mL and 5.31 mg/100 mL for SPB and SAGPB, respectively. A reduction in ascorbic acid on storage was observed, which has been reported to be mainly caused due to anaerobic degradation, especially in thermally preserved products [20]. Similar reduction in vitamin C content has also been reported in other fruit juices such as orange juices [21]. A gradual reduction on storage led to the vitamin C content becoming 74% lower after 90 d and 79% lower after 150 d of its initial value in SPB samples. However, in SAGPB, the reduction was lower (56% lower on 90 d and 61% lower on 150 d than the initial value). Aloe gel is reported to have good antioxidant activity [22] and, hence, could have protected vitamin C from oxidation to some extent during storage.

The content of total polyphenols in the beverages was found to be 235 mg/100 mL and 240 mg/100 mL for SPB and SAGPB, respectively, on the initial day. Polyphenol content of both the beverages was found to initially increase on storage up to 45 days in SPB and 90 days for SAGPB, after which there was a decrease. The flavonoid content showed similar trend, that is, increases up to 45 days of storage, followed by decrease. After 150 days of storage, SAGPB demonstrated 15.6% and 23% higher polyphenol and flavonoid contents, respectively, significantly higher than SPB.

Very little information is available in literature regarding the phenolic content of papaya RTS beverage on storage. The increase in phenolic content on storage could be due to the release of the free acids from their bound forms. Similar observations have also been reported for some individual phenolic compounds on storage. In a study by Klimczak et al. [21], an increase in the concentration of p-coumaric acid and ferulic acids was observed during 6-months storage at 18, 28, and 38°C in orange juice. The same study also reported an increase in concentration of sinapic acid for 4 months after which there was a decrease, a trend similar to the one observed in the present study (i.e. initial increase followed by decrease). Other studies also report an increase in concentration of phenolic compounds, particularly p-coumaric acid and ferulic acids on storage [23, 24]. Since changes in individual phenolic constituents contribute to the overall phenolic content of the beverages, the results observed in the present study could be compared to those reported in literature for individual phenolic compounds.

In the present study, the spectrophotometric method using Folin-Ciocalteu reagent was employed to determine the total phenols as it is a routine analytical tool used widely for estimating differences in polyphenols among fruits, vegetables, and their products [25]. However, this method lacks sensitivity [26] and, hence, could react with other reducing compounds such as carotenoids, sugars, and vitamin C [27] present in fruits and fruit products. In the present study, reducing sugars were found to increase on storage in the beverage samples.

The positive health effects of polyphenols and flavonoids have been well explored in the past decade [28]. A higher content of these phytochemicals in the Aloe gel enriched beverage indicates the beneficial role of Aloe gel as a functional ingredient in fruit based beverages.

Both the beverage samples were found to be free from microbial proliferation till the end of storage period in terms of standard plate count and yeast and mold count, thereby indicating its fitness for consumption even after 5 months of storage. This could be attributed to the effective pasteurization treatment, addition of citric acid which acts as a preservative, and also due to the addition of spices which are known to possess good antimicrobial activity [7, 8]. The combined beneficial effect of the above mentioned factors could have led to the higher product stability observed in both the samples.

**Figure 1:**
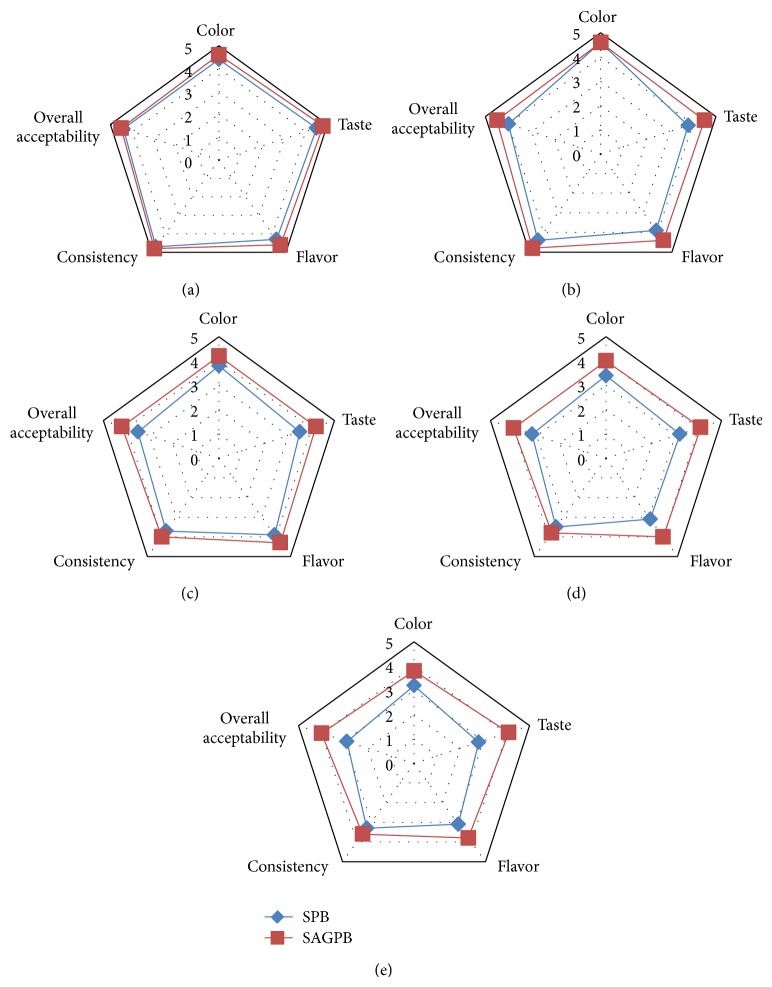
Sensory acceptability of the developed beverages—SPB (spiced papaya beverage) and SAGPB (spiced Aloe gel-papaya beverage blend) after 0 d (a), 45 d (b), 90 d (c), 120 d (d), and 150 d (e) of storage.

The results of the sensory acceptability of the SPB and SAGPB on storage have been depicted in Figure 1. No significant differences were observed between the samples initially, except in terms of taste and flavor wherein SAGPB scored higher due to its balanced taste. A reduction in sensory attributes was witnessed with the progression of storage period. Results indicated maintenance of fresh sensory quality in Aloe gel enriched beverage for a longer period compared to control beverage. End of storage was marked by significant differences between the samples for all the parameters evaluated primarily the taste and flavor. The SPB beverage scored lower in taste and flavor mainly due to the sour/acidic taste which increased on storage. This could be evidenced in the chemical analysis results which revealed SPB to have higher acidity value. On the other hand, the SAGPB sample maintained the balanced taste with only slight change in the acidity thereby recording higher scores for taste and flavor. The overall acceptability of SAGPB was significantly higher (4) compared to SPB (2.9) at the storage period, demonstrating the beneficial effect of Aloe gel addition on maintaining the sensory quality of the developed beverage.

## 4. Conclusions

This present study revealed various beneficial effects of Aloe gel incorporation to spiced papaya beverage. The effects include better product quality characteristics (physicochemical, sensory, and microbial quality), enhanced phytochemical profile, and improved storage stability. Aloe gel could thus be used to deliver natural bioactive phytochemicals to formulate functional fruit beverages. Aloe-fruit beverage blends could also offer an attractive means of increasing the consumption of unpalatable Aloe gel/juice. The developed Aloe gel-fruit based spiced functional beverage blend could be promoted as a nutraceutical product with multiple benefits to the consumers.

## Figures and Tables

**Table 1:** Sensory acceptability of spiced papaya—Aloe gel beverage (SAGPB) blends.

Sample	Color	Taste	Flavor	Consistency	Overall acceptability
0% SAGPB/SPB	4.57 ± 0.39^b^	4.41 ± 0.36^b^	4.35 ± 0.44^c^	4.85 ± 0.22^a^	4.35 ± 0.41^c^
10% SAGPB	4.58 ± 0.44^b^	4.51 ± 0.34^b^	4.58 ± 0.34^b^	4.75 ± 0.38^a^	4.58 ± 0.44^b^
20% SAGPB	4.71 ± 0.46^a^	4.71 ± 0.36^a^	4.71 ± 0.24^a^	4.64 ± 0.22^a^	4.64 ± 0.34^a^
30% SAGPB	4.78 ± 0.36^a^	4.71 ± 0.36^a^	4.85 ± 0.22^a^	4.78 ± 0.24^a^	4.71 ± 0.36^a^

Mean ± SD with different superscripts in each column differ significantly (DMRT test, *P* < 0.05).

**Table 2:** Quality characteristics of the developed functional beverages.

Sample	Storage period (days)	Titratable acidity (%)	Total soluble solids (°Brix)	Total sugars (mg/100 mL)	Reducing sugars (mg/100 mL)	*L* ^*^	*a* ^*^	*b* ^*^	Nonenzymatic browning (OD, 440 nm)
SPB	0	0.27 ± 0.04^f^	15.6 ± 0.41^d^	16.7 ± 0.6^d^	6.7 ± 0.2^e^	45.6 ± 1.16^a^	1.63 ± 0.12^b^	9.23 ± 0.28^d^	0.13 ± 0.01^f^
	45	0.37 ± 0.03^e^	16.4 ± 0.55^c^	21.6 ± 0.8^c^	8.6 ± 0.2^c^	43.2 ± 1.21^b^	1.36 ± 0.21^d^	8.83 ± 0.40^e^	0.23 ± 0.01^e^
	90	0.49 ± 0.04^d^	15.9 ± 0.25^c^	25.7 ± 1.4^b^	10.3 ± 0.4^a^	44.5 ± 0.73^a^	0.91 ± 0.08^e^	8.10 ± 0.41^f^	0.51 ± 0.02^c^
	120	0.58 ± 0.03^b^	18.4 ± 0.3^a^	27.8 ± 1.4^a^	10.6 ± 0.4^a^	42.1 ± 0.81^b^	0.69 ± 0.21^e^	7.91 ± 0.08^f^	0.71 ± 0.04^b^
	150	0.62 ± 0.01^a^	18.7 ± 0.25^a^	29.1 ± 1.1^a^	11.1 ± 0.3^a^	41.2 ± 0.52^b^	0.61 ± 0.14^e^	7.83 ± 0.26^f^	0.88 ± 0.04^a^

SAGPB	0	0.27 ± 0.04^f^	15.7 ± 0.25^d^	17.2 ± 0.3^d^	6.9 ± 0.1^e^	46.4 ± 0.66^a^	2.36 ± 0.33^a^	11.9 ± 0.37^a^	0.14 ± 0.01^f^
	45	0.32 ± 0.02^e^	17.6 ± 0.41^b^	18.2 ± 0.7^d^	7.3 ± 0.2^d^	41.5 ± 1.02^b^	2.06 ± 0.12^b^	12.3 ± 0.69^a^	0.16 ± 0.04^f^
	90	0.41 ± 0.02^a^	16.3 ± 0.25^c^	21.7 ± 0.7^c^	8.7 ± 0.2^c^	45.1 ± 0.36^a^	1.71 ± 0.26^c^	11.4 ± 0.75^b^	0.31 ± 0.07^e^
	120	0.48 ± 0.01^d^	17.1 ± 0.11^b^	23.2 ± 1.3^c^	9.0 ± 0.2^c^	44.5 ± 0.33^a^	1.53 ± 0.24^c^	10.3 ± 0.06^c^	0.47 ± 0.04^d^
	150	0.54 ± 0.02^c^	17.4 ± 0.21^b^	24.5 ± 1.5^b^	9.5 ± 0.3^b^	44.1 ± 0.12^a^	1.31 ± 0.21^d^	9.31 ± 0.17^d^	0.57 ± 0.02^c^

Mean ± SD with different superscripts in each column differ significantly (DMRT test, *P* < 0.05).

**Table 3:** Phytochemical content of the developed functional beverages.

Sample	Storage period (days)	Vitamin C (mg/100 mL)	Total polyphenols (mg/100 mL)	Total flavonoids (mg/100 mL)
SPB	0	5.22 ± 0.11^a^	235 ± 5.00^b^	114 ±1.50^c^
	45	3.65 ± 0.17^c^	273 ± 2.50^a^	120 ± 1.25^c^
	90	1.35 ± 0.05^f^	240 ± 5.00^b^	110 ± 0.75^c^
	120	1.25 ± 0.05^f^	216 ± 5.50^c^	93 ± 1.23^d^
	150	1.08 ± 0.11^f^	198 ± 4.02^d^	91 ± 1.15^d^

SAGPB	0	5.31 ± 0.12^a^	240 ± 10.2^b^	115 ± 1.51^c^
	45	4.62 ± 0.19^b^	290 ± 0.40^a^	138 ± 2.85^a^
	90	2.35 ± 0.11^d^	310 ± 5.00^a^	127 ± 2.75^c^
	120	2.19 ± 0.09^d^	267 ± 5.55^a^	116 ± 1.45^c^
	150	2.05 ± 0.12^e^	229 ± 4.55^b^	112 ± 1.55^c^

Mean ± SD with different superscripts in each column differ significantly (DMRT test, *P* < 0.05).
